# Measuring parents’ perspective on continuity of care in children with special health care needs

**DOI:** 10.5334/ijic.2202

**Published:** 2015-12-02

**Authors:** Paola Rucci, Jos Latour, Elisa Zanello, Simona Calugi, Silvia Vandini, Giacomo Faldella, Maria Pia Fantini

**Affiliations:** Division of Hygiene and Biostatistics, Department of Biomedical and Neuromotor Science – DIBINEM, Alma Mater Studiorum University of Bologna, Bologna, Italy; Faculty of Health and Human Sciences, School of Nursing and Midwifery, Plymouth University, Plymouth, UK; Faculty of Health Science, School of Nursing and Midwifery, Curtin University, Kent Street, Bentley, WA 6102, Australia; Division of Hygiene and Biostatistics, Department of Biomedical and Neuromotor Science – DIBINEM, Alma Mater Studiorum University of Bologna, Bologna, Italy; Division of Hygiene and Biostatistics, Department of Biomedical and Neuromotor Science – DIBINEM, Alma Mater Studiorum University of Bologna, Bologna, Italy; Neonatology and Neonatal Intensive Care Unit, Department of Medical and Surgical Sciences, S.Orsola Malpighi Hospital, University of Bologna, Bologna, Italy; Neonatology and Neonatal Intensive Care Unit, Department of Medical and Surgical Sciences, S.Orsola Malpighi Hospital, University of Bologna, Bologna, Italy; Division of Hygiene and Biostatistics, Department of Biomedical and Neuromotor Science – DIBINEM, Alma Mater Studiorum University of Bologna, Bologna, Italy

**Keywords:** special health care needs, children with preterm birth, factor analysis, integrated care, family support, parents’ experience

## Abstract

**Introduction:**

Children with special health care needs are an exponentially growing population needing integrated health care programmes that involve primary, community, hospital and tertiary care services. The aims of the study are (1) to develop and validate the Special Needs Kids Questionnaire (SpeNK-Q) designed to measure parents’ perspective on continuity of care for children with special health care needs and (2) to evaluate the continuity of care based on parental experiences in this population.

**Methods:**

SpeNK-Q was derived from a previous qualitative study and was based on Haggerty’s constructs of informational, management and relational continuity. Parents of preterm birth children completed the 20-item SpeNK-Q at the second or subsequent planned follow-up visit after the child’s hospital discharge. Principal component analysis was used to examine the structure of the instrument.

**Results:**

Principal component analysis of 101 questionnaires administered allowed us to identify five factors explaining 60.2% of item variance: informational continuity; coordination of care; continuity of family–paediatrician relationship; family support; information on care plan.

**Conclusions and discussion:**

SpeNK-Q proved to be a psychometrically promising instrument. Its utilisation could improve the identification of areas for service development, the delivery of coordinated care and support policy makers in redesigning integrated services.

## Background

Children with special health care needs are a highly vulnerable subset of the child population [1]. According to the definition of the Maternal and Child Health Bureau, children with special health care needs are those who ‘have, or are at an increased risk for, a chronic physical, developmental, behavioral, or emotional conditions and who also require health and related services of a type or amount beyond that generally required by children’ [2] (p. 138).

The prevalence of non-institutionalised children with special health care needs aged 0–17 has been estimated as 12% in 1999–2000 in USA [3] and has been growing exponentially in the past decades due to novel treatments in life-threatening paediatric conditions that increase the survival of children with serious congenital or acquired diseases. This success factor within paediatrics has considerable societal costs and important financial and organisational consequences for health care planning [4].

Low birth weight newborns (<2500 g) constitute about 6% of all newborns [5]. Very low birth weight (<1500 g) infants are at increased risk of chronic conditions and of poor neurodevelopment and can be considered a specific subgroup of children with special health care needs [2]. In high-income countries, progress in medical care has led to improved survival and long-term outcome among preterm infants with very low birth weight, but considerable risks for child health and development remain a matter of concern [6,7].

Similar to adult patients, children with chronic or complex health conditions require the implementation and coordination of a variety of health care services and providers at different levels, from primary care to hospital care, over an extended period of time. In this context, continuity of care, meaning the degree to which the patients experience their perceived care over time as coherent [8], represents a key element of health care provision. In recent years, continuity of care has received more attention as a result of changes in health care systems, due to the increase in patients with chronic and multiple diseases and the increasing complexity of the health care services [9]. Despite the recognised importance of continuity of health care for patients with chronic or complex conditions, the main research focus has been on adults and elderly, while less attention has been paid to children with special health care needs and their families.

To assess continuity of care for these children, the critical role of parents, mediating between the child’s needs and health care services and professionals, must be taken into account [10]. Parents should be involved in the assessment in order to measure and improve continuity of care for their children. Recently, Patient Reported Experience Measures have garnered attention for measuring experience of patients interacting with an array of professionals and services within a complex health care system. Patient Reported Experience Measures proved to provide more information than patient satisfaction questionnaires by encouraging the users to describe their actual experience of the care received [11]. A review of the instruments measuring continuity of care showed that most available instruments on continuity of care from patients’ perspective are designed to assess this construct in specific adult populations and settings, such as patients with diabetes, cancer, mental health problems, previously hospitalised patients, complex and chronic diseases, people being treated in primary care settings or patients in general regardless of morbidity or care setting [12]. To our knowledge, only one questionnaire has been developed to measure continuity of care in child population from the family’s perspective, but it applies only to mental health care [13].

Recently we performed a qualitative study examining the perceptions and experiences of parents of children with special health care needs while interacting with various health care services and providers [14]. Continuity of care was found to be important to parents, and several key elements were useful to develop a quantitative measure of this construct.

The conceptual framework underlying our instrument development refers to the definitions of continuity of care provided by Haggerty and colleagues [15]. This refers to three types of continuity of care. ‘Informational continuity of care’ addresses ‘the use of information on past events and personal circumstances to make current care appropriate for each individual’ (p. 1220) among providers and among health care events. ‘Management continuity of care’ addresses ‘a consistent and coherent approach to the management of a health condition that is responsive to a patient’s changing needs’ (p. 1220), which is especially important for in chronic or complex clinical diseases. ‘Relational continuity of care’ refers to ‘an ongoing therapeutic relationship between a patient and one or more providers’ (p. 1220), which bridges past to current care and provides a link to future care [15]. Valid measures of continuity of care for children with special health care needs must involve parents in order to identify the areas of improvement potential and gaps in care coordination from user’s perspective, in a systematic and reliable way, The aims of the present study were to develop and validate an instrument to measure continuity of care for children with special needs from the perspective of parents with preterm infants and to evaluate the continuity of care in the population assessed.

## Materials and Methods

### Setting

The study was performed at the University Hospital of Bologna (St. Orsola-Malpighi) in the Preterm Infant Follow-up/Day-Hospital Clinic of the Neonatology Unit. For the preterm infants the Unit activates a standardised follow-up procedure at the Clinic, after hospital discharge. The follow-up procedure includes planned visits from 3 until 42 months of the child’s corrected age (every 3 months in the first year, every 6 months in the second year and every 12 months later). Additional visits may be scheduled for any further clinical needs.

### Participants

Study participants were recruited from parents of children with preterm birth requiring integrated health care programmes at the Preterm Infant Follow-up/Day-Hospital Clinic of the Neonatology Unit. Inclusion criteria were: (i) access to the Preterm Infant Follow-up/Day-Hospital Clinic of the Neonatology Unit for the second or subsequent follow-up visit of the child and (ii) adequate level of knowledge of Italian language. The ascertainment of inclusion criteria was made by the Preterm Infant Follow-up/Day-Hospital Clinic of the Neonatology Unit personnel, who invited the eligible parents to participate in the study. Parents at the first follow-up visit (3 months of corrected child’s age) and parents who were not sufficiently fluent in the Italian language were excluded. All parents meeting inclusion criteria accepted participation in the study.

Eighty-one parents of 101 children with preterm birth were recruited during a 4-month period (November 2013–March 2014) and completed the questionnaire.

The Ethics Committee of the Bologna University Hospital Authority approved the study procedures and all parents consented to participate in the study.

### Instrument development

The Special Needs Kids (SpeNK) Questionnaire (SpeNK-Q) was developed in the framework of the SpeNK study [14]. The Emilia-Romagna Region SpeNK Project was designed to describe the implementation of existing sheltered hospital discharge procedures and integrated clinical pathways for children with complex or chronic health conditions and special health care needs and to assess the family’s perspective on continuity of care and the role of family paediatrician. The ‘sheltered’ discharge is a specific hospital procedure for children with complex social and/or health care needs that includes the activation of community services and primary care providers, who take care of the child after hospital discharge.

SpeNK-Q was derived from the results of the SpeNK-I qualitative study [14] and was based on Haggerty’s constructs of informational, management and relational continuity [15,16]. In the SpeNK-I study, 16 families of children with special health care needs were interviewed to explore their experiences and perceptions on informational, management and relational continuity of care from hospitalisation to the first months after discharge to the home. We found that the three domains of continuity of care were relevant to parents, with different key elements related to the treatment phase (i.e. hospitalisation, discharge, after discharge) [14].

The item development of the questionnaire was carried out through several steps. First, we reviewed the literature about measures of continuity of care [9] and found that no measures for continuity of care specific for children. Thus, we chose to refer to Haggerty’s generic measure of continuity of care and Miller’s study [10,15,16] to generate item statements about continuity of care for children from parents’ perspective. Second, we adapted 36 items from Haggerty’s generic measure about care received by adult patients to parents’ perspective on their child’s care and to the Italian health care organisational context. Third, we selected the final 20 item statements, by retaining the items which occurred most frequently in parents’ narratives about continuity of care in SpeNK-I Study [14]. Lastly, we attributed to each of the 20 SpeNK-Q item statements a 5-point response option, to measure agreement or frequency. We decided to use a 5-point Likert-type scale because, using four response categories, people who see both positive and negative aspects of their perceptions would be forced to lean either towards the positive or the negative; ‘uncertain’ would give them an option they feel comfortable with. There is also some evidence that the absence of a mid-point on an importance scale produces distortions in the results obtained. It has been reported that the lack of a mid-point has resulted in more negative ratings than would be achieved when a mid-point was available [17].

The item statements explore parents’ perspective about their relationship and interactions with: (1) the family paediatrician (knowledge of the child’s medical history, partnership and confidence); (2) the main coordinator (knowledge of the child’s health needs, continuity with other providers, services and clinicians); (3) the network of health care providers and services involving child care such as care provision, coherence and availability of information, parents’ involvement and engagement, knowledge of the child, experiences of receiving advice, and health care systems. SpeNK-Q includes two open questions aiming at identifying: (1) the person who is in charge of most of the child’s health; (2) the person who coordinates the child’s health care (i.e. main coordinator: for example, Preterm Infant Follow-up/Day-Hospital Clinic of the Neonatology Unit physician, family paediatrician, nurse, etc.). The two questions were used to facilitate understanding of the following items and were not included in the analysis (Appendix).

### Statistical analysis

Principal component analysis with orthogonal (varimax) and oblique (promax) rotation was used to analyse the construct validity of the instrument [18]. Kaiser-Meier-Olkin was used to assess the sampling adequacy. The sampling was considered adequate if Kaiser-Meier-Olkin was higher than 0.5.

The number of questionnaires to be administered was determined in advance as *N* = 100, to ensure a 5:1 subject to item ratio, as recommended for principal component analysis [19]. We used the child as the unit of analysis.

The number of factors to be extracted was defined by inspecting the scree plot and considering their interpretability and consistency with the criteria that guided the construction of the instrument.

After determining the number of factors, Cronbach’s alpha was calculated for each factor to evaluate the internal consistency. Cronbach’s alpha was assumed to be satisfactory when it was ≥0.70 [20]. We computed the factor scores using the regression method [21]. These scores are expressed as *Z* scores (mean = 0, standard deviation = 1) and are an estimate of the score each subject would have on each factor, if it were measured directly.

Because the principal component analysis is based on the assumption that items are continuous variables with a normal distribution and that observations are independent, we took the log-transform of the variables and replicated the principal component analysis using Mplus 7 software that includes analytic procedures suitable for ordinal-level variables, with a skewed distribution, and for non-independent observations (twins). Factors were estimated using a robust weighted least squares estimator.

Using Mann–Whitney test we assessed the association between clinical characteristics of the children (i.e. clinical complications, birth weight <1500 g, intensity of the health care services received, parity) and the factor scores of the SpeNK-Q.

To take into account the presence of twins, we also conducted secondary mixed effects analyses in which factors were regressed on children characteristics and children were nested into their family.

We calculated the percentage of parents responding to the answer options ‘disagree and strongly disagree’ or ‘never and sometimes’ in order to identify lower levels of continuity of care according to parents’ perspective.

The significance level was set at *p* < 0.05. IBM SPSS Statistics (version 20, Chicago, USA) and Mplus Version 7 (http://www.statmodel.com) were used for the analyses.

## Results

Eighty-one parents of 101 children with preterm birth (including 20 twins) participated in the study. Parents of twins completed one questionnaire for each child. The total number of completed questionnaires was 101. The SpeNK-Q took about 10 min to be completed by parents and was acceptable and easy to administer.

Parents’ and children’s characteristics are presented in Table 1. Over half of the parents were mothers, with a mean age of 34.2 (±6.3; range: 18–51) years. Children were female in 52.5% (*n* = 53) of cases, had a mean gestational age of 30.1 (±2.3, range 23.1–35.3) weeks and a mean birth weight of 1280.6 (±352.9, range: 498–2499) g. At the time of SpeNK-Q administration, children had a mean age of 20.7 (±9.9; range: 6–43) months and had been discharged from the hospital about 19 months before. The questionnaire was completed by parents of 32 (31.7%) children within one year from hospital discharge and by parents of 69 (68.3%) children one year after hospital discharge.

The principal component analysis was carried out with varimax and promax rotation. Kaiser-Meier-Olkin was 0.64, indicating that the 20 items of the SpeNK-Q were appropriate for principal component analysis. By inspecting the scree plot, a change in the curvature was observed after the sixth factor, suggesting that six factors were sufficient to summarise the variance of the items in a parsimonious way and that the subsequent factors were nuisance factors. After comparing the five and sixfactor solutions, a five-factor varimax (orthogonal) solution was selected as the best in terms of interpretability. This solution accounted overall for 60.2% of item variance. The first factor identified was (1) ‘informational continuity’ that included seven items and accounted for 21.4% of the variance, followed by (2) ‘care coordination’ with four items accounting for 12.3% of the variance, (3) ‘continuity of family–paediatrician relationship’ with three items accounting for 10.4% of the variance, (4) ‘family support’ with four items accounting for 8.7% of the variance and (5) ‘information on care plan’ with two items accounting for 7.4% of the variance. All item loadings were greater than 0.47, except for the item 8 (loading 0.31) (Table 2).

Cronbach’s alpha for each factor is included in Table 2. Values were adequate (>0.70), except for the factor ‘family support’ (0.63).

**Figure 1.**
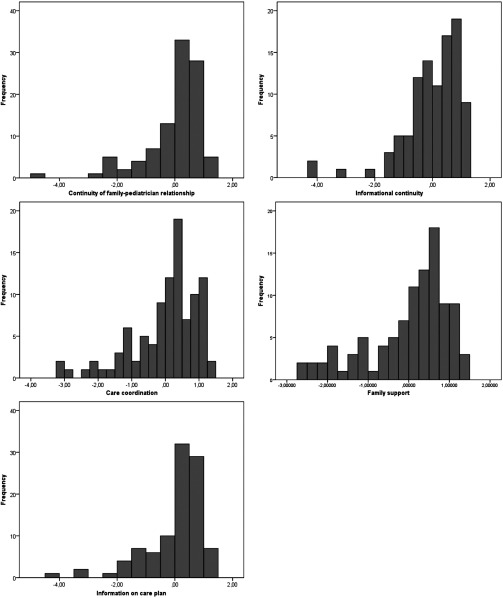
Frequency distribution of factor scores.

The distribution of factor scores is shown in Figure 1. Each factor showed a sufficient variability, confirming the ability to discriminate between high and low levels of continuity of care.

SpeNK-Q factor scores were unrelated with clinical characteristics and intensity of care received by the children in univariate and multivariate analyses, and in multilevel analyses adjusted for the presence of twins.

When principal component analysis was replicated on log-transformed variables, using an estimation method suitable for ordinal-level variables and taking into account the presence of twins in the sample, results were unchanged, and the factor structure and items loadings were the same (results not reported).

Lastly, we calculated the percentages of the item responses to identify areas with different levels of continuity of care according to parents’ perspective (Table 3). Items endorsed with the lowest frequency were ‘the main coordinator contacts other clinicians about health care received by their child’ (61.6%) and ‘he/she keeps in contact with parents even when the child receives health care by others’ (35.4%). Furthermore, over 70% of the parents reported that they had to provide the results of a specialist’s visit to the person who was seeing their child. Over 20% of the parents indicated that the people who took care of their child told them different things about his/her health and over 40% had to repeat information about their child’s health that should have been in his/her medical record.

## Discussion

To ensure continuity of care and to identify gaps in care coordination for children with special health care needs, it is essential to develop valid measures for the assessment of perceptions and experiences of parents interacting with multiple services and providers that are involved in their child’s care. The SpeNK-Q proved to be a psychometrically promising instrument to measure continuity of care in children with special health care needs and easy to administer to parents. It may facilitate the identification of improvement potential into care for these children and help reduce the risk of fragmentation and discontinuity within the health care pathway.

The five SpeNK-Q factors identified encompassed several relevant aspects of the continuity of care and a broad spectrum of information related with the parents’ perspective. In particular, Factor 1 ‘informational continuity’ focused on the consistency of the information shared between clinicians and the feeling of being ‘well known’ versus ‘abandoned’ by health care providers. The items referred to the experience of a ‘common thread linking care from one provider to another and from one health care event to another’ (i.e. informational continuity) [15]. Only item 8 had low factor loading and could be considered for removal.

The Factor 2 ‘care coordination’ was related to the role played by the care coordinator, identified by the parent as the professional who is in charge of most of the child’s health care. It consisted of items assessing how well the coordinator knows all health care needs, maintains regular contact with the family of children and with other clinicians and is updated about care provided by other clinicians. This factor was consistent with the ‘coordinator role’ dimension of the questionnaire ‘Patient Perceived Continuity from Multiple Clinicians’ developed and validated by Haggerty et al. [16].

Items composing Factor 3 ‘continuity of family–paediatrician relationship’ reflected the experience of an ongoing therapeutic relationship between the child and the family paediatrician. The fact that we identified these two factors, reflecting the informational continuity, on the one hand, and the relational continuity, on the other hand, is consistent with recent studies about continuity of care [9,22]. Aller and colleagues [9] underlined the distinction between relational continuity, referring to the patient–provider relationship, and ‘seamless care’ considered as continuity across care levels, which includes both transfer of medical information and care coherence.

The Factor 4 ‘family support’ concerned the information given to the family about the child’s conditions, for taking care of the child at home, coping with minor complications and the possibility of getting answers or advice quickly when necessary. Recently we published a study on parents’ experiences and perceptions of the continuity of care provided to their children with special health care needs after hospital discharge [14]. We found that, according to parents, the support received through the information and training provided by health care professionals was essential to make them able to care for their children. It was crucial to help parents cope with the transition from the hospital setting to the new responsibilities connected with the home care of their child.

The Factor 5 ‘Information on care plan’ concerned the explanations given to the family about the care provided and planned for the child such as treatments, plan of tests and examinations. This is a new and specific dimension, distinct from the ‘informational’ continuity of care, and deserves further investigations to examine whether it represents an independent aspect of the continuity of care.

The five SpeNK-Q factors encompassed different issues compared with the other unique instrument measuring the continuity of care in child population developed by Tobon et al. [13]. This difference could be explained by the diverse demographic characteristics (i.e. newborns vs. adolescents) and health care needs of samples (i.e. special health care needs vs. mental health), requiring a different array of services in different settings. Moreover, Tobon et al. used an *a priori* approach to develop their sub-scales.

The issues addressed by SpeNK-Q factors are similar to themes emerged in our previous qualitative study [14], indicating that our instrument is able to detect significant areas of continuity of care that are relevant to parents of children with special needs in different settings, such as communication, information exchange and parent involvement in the child’s care [23]. On the contrary, we did not find consistency between our factor solution and Haggerty’s one, probably because of the differences in perspective (parents vs. adult patients) and health care organisational context.

Moreover, our data indicated that continuity of care was unrelated to the clinical characteristics of the child and the intensity of health care services received, indicating that parents perceive a high level of continuity of care regardless of the severity of the child’s condition.

The analysis of item responses underscores some issues that could be taken into account in order to improve continuity of care for children with special health care needs. We found that the main area of improvement concerned the role of the care coordinator. In fact, about 40% of parents stated that the main coordinator had poor or no attention in contacting other clinicians about health care received by the child and that often he/she did not keep in contact with parents when the child received health care by others professionals. Furthermore, management/informational continuity seemed to be a weakness in the continuity of care perceived by our families because almost 75% of parents indicated that they have to provide, often or always, the report of a specialist’s visit to the person who was seeing their child, that they had to repeat information about the child’s health which should have been in his/her medical record and that the people who took care of the their child told different things about his/her health. To our knowledge, at present there are no other available quantitative studies investigating areas of discontinuity of care for children with special health care needs from parents’ perspective. Therefore, these areas should be taken into account by the health care providers to improve continuity of care in this specific setting and population.

The main strength of this study is that SpeNK-Q is the first instrument measuring continuity of care provided to children with special health care needs from the parents’ perspectives. Moreover, our study includes parents with different duration of the experience of care, thus increasing the sample variability as regards a core element of continuity of care, i.e. patient’s experience of care over time [8].

The study has some limitations to address. The first is the generalisability and utility of our instrument to assess continuity of care of children with chronic conditions or special health care needs other than preterm birth. The second limitation relates to the lack of information about test–retest reliability. We decided not to administer the questionnaire to the same participant at two different times to avoid burdening families who were living in difficult situations. The third limitation concerns the inability to assess the concurrent validity with other existing instruments because no validated instruments assessing continuity of care in children are available in Italian language. The fourth is the limited sample size that did not allow to use confirmatory factor analysis or item response theory analysis to examine the performance of items in deeper detail [24,25].

## Conclusion

The SpeNK-Q proved to be a promising instrument encompassing multifaceted components of continuity of care, which could be integrated in routine practice to assess the users’ experience of different health care models and procedures. Thus, the SpeNK-Q may be used to identify areas of improvement from users’ perspective to be integrated with professionals’ and systems’ viewpoints [26]. This could represent a first step towards an experience-based design approach in a public health perspective, by making the user integral to the process of redesigning services [27].

Further studies are needed to evaluate the test–retest reliability of the instrument, to analyse the item response in large samples, to confirm the factor structure and extend the psychometric properties of the SpeNK-Q in children with other special health care needs recruited from other national and international settings.

## Figures and Tables

**Table 1.**
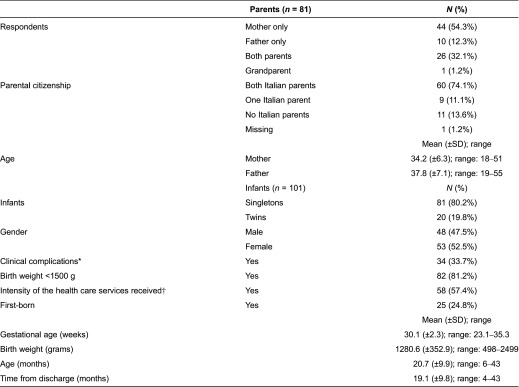
Characteristics of parents (*n* = 81) and infants (*n* = 101)

*At least one complication during the hospitalisation at birth.

†Presence of at least one of the following: sheltered discharge; more than three follow-up visits; at least one re-hospitalisation.

**Table 2.**
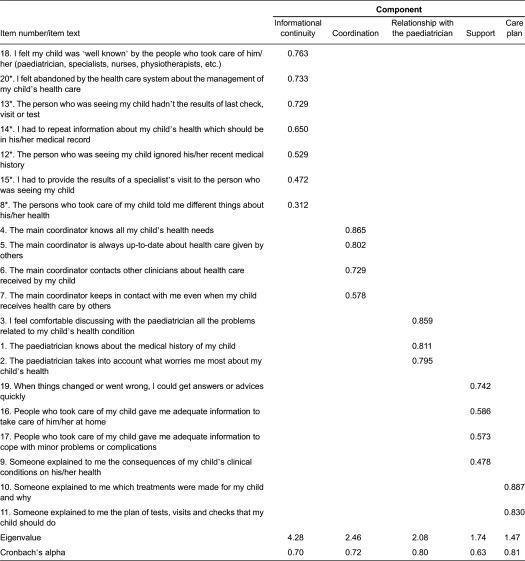
Principal component analysis with orthogonal rotation

*Reverse scored item, calculated by subtracting the item score from 6.

**Table 3.**
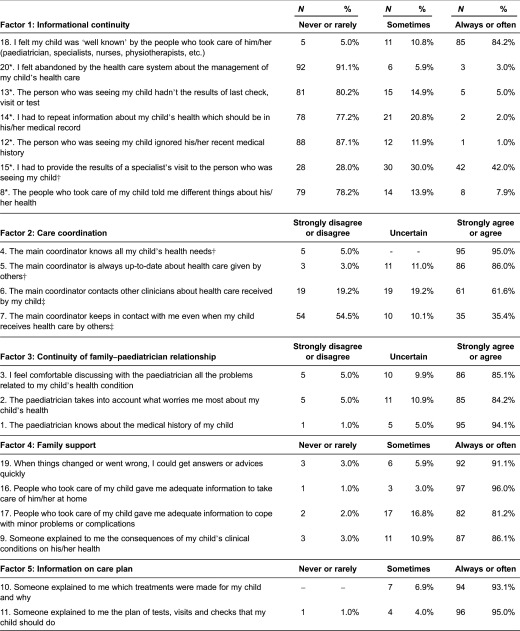
Responses to the SpeNK-Q item statements

*Reverse scored item, calculated by subtracting the item score from 6.

†Missing data *n* = 1.

‡Missing data *n* = 2.
